# Supplementary material for: A method for high precision sequencing of near full-length 16S rRNA genes on an Illumina MiSeq
Source: PeerJ. 2016 Sep 20;4:e2492. doi: 10.7717/peerj.2492 (PMC5036073; doi:10.7717/peerj.2492)
Supplement: Supplemental Information 1 [file peerj-04-2492-s001.docx]

**Table S1:** Primer sequences used in this study

| **Primer name** | **Sequence** |
| --- | --- |
| Long_forward_1 | ACACTCTTTCCCTACACGACGCTCTTCCGATCTNNNNNNNNNNGTTGGCCGCGAGAGTTTGATCMTGGCTCAG |
| Long_forward_2 | ACACTCTTTCCCTACACGACGCTCTTCCGATCTNNNNNNNNNNTATTAACTNCGAGAGTTTGATCMTGGCTCAG |
| Long_forward_3 | ACACTCTTTCCCTACACGACGCTCTTCCGATCTNNNNNNNNNNCTAATGGCNNCGAGAGTTTGATCMTGGCTCAG |
| Long_forward_4 | ACACTCTTTCCCTACACGACGCTCTTCCGATCTNNNNNNNNNNAACCAGTCNNNCGAGAGTTTGATCMTGGCTCAG |
| Long_forward_5 | ACACTCTTTCCCTACACGACGCTCTTCCGATCTNNNNNNNNNNGAACGGAGCGAGAGTTTGATCMTGGCTCAG |
| Long_forward_6 | ACACTCTTTCCCTACACGACGCTCTTCCGATCTNNNNNNNNNNACTGAAGTNCGAGAGTTTGATCMTGGCTCAG |
| Long_forward_7 | ACACTCTTTCCCTACACGACGCTCTTCCGATCTNNNNNNNNNNTTGGCTATNNCGAGAGTTTGATCMTGGCTCAG |
| Long_forward_8 | ACACTCTTTCCCTACACGACGCTCTTCCGATCTNNNNNNNNNNTGGCGATTNNNCGAGAGTTTGATCMTGGCTCAG |
| Long_forward_9 | ACACTCTTTCCCTACACGACGCTCTTCCGATCTNNNNNNNNNNCCTCTGATCGAGAGTTTGATCMTGGCTCAG |
| Long_forward_10 | ACACTCTTTCCCTACACGACGCTCTTCCGATCTNNNNNNNNNNCTCATGCGNCGAGAGTTTGATCMTGGCTCAG |
| Long_forward_11 | ACACTCTTTCCCTACACGACGCTCTTCCGATCTNNNNNNNNNNTTCAGCGANNCGAGAGTTTGATCMTGGCTCAG |
| Long_forward_12 | ACACTCTTTCCCTACACGACGCTCTTCCGATCTNNNNNNNNNNGGATGCCANNNCGAGAGTTTGATCMTGGCTCAG |
| Long_forward_13 | ACACTCTTTCCCTACACGACGCTCTTCCGATCTNNNNNNNNNNCGGTCGAGCGAGAGTTTGATCMTGGCTCAG |
| Long_forward_14 | ACACTCTTTCCCTACACGACGCTCTTCCGATCTNNNNNNNNNNAAGACTACNCGAGAGTTTGATCMTGGCTCAG |
| Long_forward_15 | ACACTCTTTCCCTACACGACGCTCTTCCGATCTNNNNNNNNNNAACGCTAANNCGAGAGTTTGATCMTGGCTCAG |
| Long_forward_16 | ACACTCTTTCCCTACACGACGCTCTTCCGATCTNNNNNNNNNNGCCTACGCNNNCGAGAGTTTGATCMTGGCTCAG |
| Long_forward_17 | ACACTCTTTCCCTACACGACGCTCTTCCGATCTNNNNNNNNNNTGACTGCTCGAGAGTTTGATCMTGGCTCAG |
| Long_forward_18 | ACACTCTTTCCCTACACGACGCTCTTCCGATCTNNNNNNNNNNATTGCCGCNCGAGAGTTTGATCMTGGCTCAG |
| Long_forward_19 | ACACTCTTTCCCTACACGACGCTCTTCCGATCTNNNNNNNNNNCAACCTTANNCGAGAGTTTGATCMTGGCTCAG |
| Long_forward_20 | ACACTCTTTCCCTACACGACGCTCTTCCGATCTNNNNNNNNNNGGAGGCTGNNNCGAGAGTTTGATCMTGGCTCAG |
| Long_forward_21 | ACACTCTTTCCCTACACGACGCTCTTCCGATCTNNNNNNNNNNAATCGATACGAGAGTTTGATCMTGGCTCAG |
| Long_forward_22 | ACACTCTTTCCCTACACGACGCTCTTCCGATCTNNNNNNNNNNACCAATTGNCGAGAGTTTGATCMTGGCTCAG |
| Long_forward_23 | ACACTCTTTCCCTACACGACGCTCTTCCGATCTNNNNNNNNNNCCTAATAANNCGAGAGTTTGATCMTGGCTCAG |
| Long_forward_24 | ACACTCTTTCCCTACACGACGCTCTTCCGATCTNNNNNNNNNNGGATTAGGNNNCGAGAGTTTGATCMTGGCTCAG |
| Long_forward_25 | ACACTCTTTCCCTACACGACGCTCTTCCGATCTNNNNNNNNNNGCGTTACCCGAGAGTTTGATCMTGGCTCAG |
| Long_reverse_1 | CTCGGCATTCCTGCTGAACCGCTCTTCCGATCTNNNNNNNNNNGTTGGCCGTAGACGGGCGGTGTGTRCA |
| Long_reverse_2 | CTCGGCATTCCTGCTGAACCGCTCTTCCGATCTNNNNNNNNNNTATTAACTNNNTAGACGGGCGGTGTGTRCA |
| Long_reverse_3 | CTCGGCATTCCTGCTGAACCGCTCTTCCGATCTNNNNNNNNNNCTAATGGCTAGACGGGCGGTGTGTRCA |
| Long_reverse_4 | CTCGGCATTCCTGCTGAACCGCTCTTCCGATCTNNNNNNNNNNAACCAGTCNNNTAGACGGGCGGTGTGTRCA |
| Long_reverse_5 | CTCGGCATTCCTGCTGAACCGCTCTTCCGATCTNNNNNNNNNNGAACGGAGTAGACGGGCGGTGTGTRCA |
| Long_reverse_6 | CTCGGCATTCCTGCTGAACCGCTCTTCCGATCTNNNNNNNNNNACTGAAGTNNTAGACGGGCGGTGTGTRCA |
| Long_reverse_7 | CTCGGCATTCCTGCTGAACCGCTCTTCCGATCTNNNNNNNNNNTTGGCTATNNNTAGACGGGCGGTGTGTRCA |
| Long_reverse_8 | CTCGGCATTCCTGCTGAACCGCTCTTCCGATCTNNNNNNNNNNTGGCGATTTAGACGGGCGGTGTGTRCA |
| Long_reverse_9 | CTCGGCATTCCTGCTGAACCGCTCTTCCGATCTNNNNNNNNNNCCTCTGATNTAGACGGGCGGTGTGTRCA |
| Long_reverse_10 | CTCGGCATTCCTGCTGAACCGCTCTTCCGATCTNNNNNNNNNNCTCATGCGNNTAGACGGGCGGTGTGTRCA |
| Long_reverse_11 | CTCGGCATTCCTGCTGAACCGCTCTTCCGATCTNNNNNNNNNNTTCAGCGANTAGACGGGCGGTGTGTRCA |
| Long_reverse_12 | CTCGGCATTCCTGCTGAACCGCTCTTCCGATCTNNNNNNNNNNGGATGCCANNTAGACGGGCGGTGTGTRCA |
| Long_reverse_13 | CTCGGCATTCCTGCTGAACCGCTCTTCCGATCTNNNNNNNNNNCGGTCGAGNTAGACGGGCGGTGTGTRCA |
| Long_reverse_14 | CTCGGCATTCCTGCTGAACCGCTCTTCCGATCTNNNNNNNNNNAAGACTACNNNTAGACGGGCGGTGTGTRCA |
| Long_reverse_15 | CTCGGCATTCCTGCTGAACCGCTCTTCCGATCTNNNNNNNNNNAACGCTAATAGACGGGCGGTGTGTRCA |
| Long_reverse_16 | CTCGGCATTCCTGCTGAACCGCTCTTCCGATCTNNNNNNNNNNGCCTACGCNTAGACGGGCGGTGTGTRCA |
| Long_reverse_17 | CTCGGCATTCCTGCTGAACCGCTCTTCCGATCTNNNNNNNNNNTGACTGCTNNTAGACGGGCGGTGTGTRCA |
| Long_reverse_18 | CTCGGCATTCCTGCTGAACCGCTCTTCCGATCTNNNNNNNNNNATTGCCGCNTAGACGGGCGGTGTGTRCA |
| Long_reverse_19 | CTCGGCATTCCTGCTGAACCGCTCTTCCGATCTNNNNNNNNNNCAACCTTANNTAGACGGGCGGTGTGTRCA |
| Long_reverse_20 | CTCGGCATTCCTGCTGAACCGCTCTTCCGATCTNNNNNNNNNNGGAGGCTGNTAGACGGGCGGTGTGTRCA |
| Long_reverse_21 | CTCGGCATTCCTGCTGAACCGCTCTTCCGATCTNNNNNNNNNNAATCGATANNTAGACGGGCGGTGTGTRCA |
| Long_reverse_22 | CTCGGCATTCCTGCTGAACCGCTCTTCCGATCTNNNNNNNNNNACCAATTGNTAGACGGGCGGTGTGTRCA |
| Long_reverse_23 | CTCGGCATTCCTGCTGAACCGCTCTTCCGATCTNNNNNNNNNNCCTAATAANTAGACGGGCGGTGTGTRCA |
| Long_reverse_24 | CTCGGCATTCCTGCTGAACCGCTCTTCCGATCTNNNNNNNNNNGGATTAGGNNTAGACGGGCGGTGTGTRCA |
| Long_reverse_25 | CTCGGCATTCCTGCTGAACCGCTCTTCCGATCTNNNNNNNNNNGCGTTACCNNNTAGACGGGCGGTGTGTRCA |
| PE_1 | AATGATACGGCGACCACCGAGATCTACACTCTTTCCCTACACGACG |
| PE_2 | CAAGCAGAAGACGGCATACGAGATCGGTCTCGGCATTCCTGCTGAACCG |
| V4_forward_1 | AATGATACGGCGACCACCGAGATCTACACAACCAGTCTATGGTAATTGTGTGCCAGCMGCCGCGGTAA |
| V4_forward_2 | AATGATACGGCGACCACCGAGATCTACACAACGCTAATATGGTAATTGTGTGCCAGCMGCCGCGGTAA |
| V4_forward_3 | AATGATACGGCGACCACCGAGATCTACACAAGACTACTATGGTAATTGTGTGCCAGCMGCCGCGGTAA |
| V4_forward_4 | AATGATACGGCGACCACCGAGATCTACACAATCGATATATGGTAATTGTGTGCCAGCMGCCGCGGTAA |
| V4_forward_5 | AATGATACGGCGACCACCGAGATCTACACACCAATTGTATGGTAATTGTGTGCCAGCMGCCGCGGTAA |
| V4_forward_6 | AATGATACGGCGACCACCGAGATCTACACACTGAAGTTATGGTAATTGTGTGCCAGCMGCCGCGGTAA |
| V4_forward_7 | AATGATACGGCGACCACCGAGATCTACACATTGCCGCTATGGTAATTGTGTGCCAGCMGCCGCGGTAA |
| V4_forward_8 | AATGATACGGCGACCACCGAGATCTACACCAACCTTATATGGTAATTGTGTGCCAGCMGCCGCGGTAA |
| V4_forward_9 | AATGATACGGCGACCACCGAGATCTACACCCTAATAATATGGTAATTGTGTGCCAGCMGCCGCGGTAA |
| V4_forward_10 | AATGATACGGCGACCACCGAGATCTACACCCTCTGATTATGGTAATTGTGTGCCAGCMGCCGCGGTAA |
| V4_forward_14 | AATGATACGGCGACCACCGAGATCTACACGAACGGAGTATGGTAATTGTGTGCCAGCMGCCGCGGTAA |
| V4_forward_16 | AATGATACGGCGACCACCGAGATCTACACGCGTTACCTATGGTAATTGTGTGCCAGCMGCCGCGGTAA |
| V4_forward_18 | AATGATACGGCGACCACCGAGATCTACACGGATGCCATATGGTAATTGTGTGCCAGCMGCCGCGGTAA |
| V4_forward_20 | AATGATACGGCGACCACCGAGATCTACACGTTGGCCGTATGGTAATTGTGTGCCAGCMGCCGCGGTAA |
| V4_forward_22 | AATGATACGGCGACCACCGAGATCTACACTGACTGCTTATGGTAATTGTGTGCCAGCMGCCGCGGTAA |
| V4_forward_24 | AATGATACGGCGACCACCGAGATCTACACTTCAGCGATATGGTAATTGTGTGCCAGCMGCCGCGGTAA |
| V4_reverse_1 | CAAGCAGAAGACGGCATACGAGATAACCAGTCAGTCAGTCAGCCGGACTACHVGGGTWTCTAAT |
| V4_reverse_7 | CAAGCAGAAGACGGCATACGAGATATTGCCGCAGTCAGTCAGCCGGACTACHVGGGTWTCTAAT |
| V4_reverse_8 | CAAGCAGAAGACGGCATACGAGATCAACCTTAAGTCAGTCAGCCGGACTACHVGGGTWTCTAAT |
| V4_reverse_9 | CAAGCAGAAGACGGCATACGAGATCCTAATAAAGTCAGTCAGCCGGACTACHVGGGTWTCTAAT |
| V4_reverse_15 | CAAGCAGAAGACGGCATACGAGATGCCTACGCAGTCAGTCAGCCGGACTACHVGGGTWTCTAAT |
| V4_reverse_16 | CAAGCAGAAGACGGCATACGAGATGCGTTACCAGTCAGTCAGCCGGACTACHVGGGTWTCTAAT |
| V4_reverse_17 | CAAGCAGAAGACGGCATACGAGATGGAGGCTGAGTCAGTCAGCCGGACTACHVGGGTWTCTAAT |
| V4_reverse_23 | CAAGCAGAAGACGGCATACGAGATTGGCGATTAGTCAGTCAGCCGGACTACHVGGGTWTCTAAT |
| V4_reverse_24 | CAAGCAGAAGACGGCATACGAGATTTCAGCGAAGTCAGTCAGCCGGACTACHVGGGTWTCTAAT |
| V4_reverse_25 | CAAGCAGAAGACGGCATACGAGATTTGGCTATAGTCAGTCAGCCGGACTACHVGGGTWTCTAAT |
| Illumina_E_1 | AATGATACGGCGACCACCGA |
| Illumina_E_2 | CAAGCAGAAGACGGCATACGA |
| V4_read_1 | TATGGTAATTGTGTGCCAGCMGCCGCGGTAA |
| V4_read_2 | AGTCAGTCAGCCGGACTACHVGGGTWTCTAAT |
| V4_index_read | ATTAGAWACCCBDGTAGTCCGGCTGACTGACT |
