# Supplementary material for: A method for high precision sequencing of near full-length 16S rRNA genes on an Illumina MiSeq
Source: PeerJ. 2016 Sep 20;4:e2492. doi: 10.7717/peerj.2492 (PMC5036073; doi:10.7717/peerj.2492)
Supplement: Supplemental Information 2 [file peerj-04-2492-s002.docx]

**Table S2:** Number of sequences per sample and OTUs obtained with closed reference OTU picking in QIIME.s

|  | Seqs. passing quality filter | | | OTUs | | | Shared OTUs | |
| --- | --- | --- | --- | --- | --- | --- | --- | --- |
| Skin  Sample | V4 | Long-Read | Long-V4 | V4 | Long-Read | Long-V4 | Long-Read & V4 | Long-V4 & V4 |
| F1.B1 | 29816 | 60 | 60 | 126 | 5 | 4 | 1 (20%) | 4 (100%) |
| F1.H | 10186 | 13 | 13 | 200 | 1 | 2 | 0 (0%) | 2 (100%) |
| F2.B2 | 6492 | 10 | 10 | 100 | 3 | 4 | 0 (0%) | 3 (75%) |
| F2.H | 5491 | 8 | 8 | 329 | 3 | 4 | 1 (33.3%) | 4 (100%) |
| F3.B2 | 5081 | 5 | 5 | 239 | 3 | 1 | 1 (33.3%) | 1 (100%) |
| F3.H | 36911 | 63 | 63 | 499 | 8 | 6 | 1 (12.5%) | 3 (50%) |
| F4.LB | 4956 | 26 | 26 | 108 | 11 | 8 | 2 (18.2%) | 8 (100%) |
| F4.LH | 1617 | 25 | 25 | 201 | 6 | 5 | 3 (50%) | 4 (80%) |
| F5.LB | 8267 | 327 | 327 | 67 | 16 | 19 | 3 (18.8%) | 14 (73.7%) |
| F5.LH | 51618 | 597 | 598 | 270 | 21 | 19 | 6 (28.6%) | 18 (94.7%) |
| F6.LB | 33644 | 311 | 311 | 215 | 7 | 8 | 1 (14.3%) | 7 (87.5%) |
| F6.LH | 21857 | 159 | 159 | 510 | 7 | 8 | 3 (42.9%) | 8 (100%) |
| Total per method | 215936 | 1604 | 1605 | 1538 | 41 | 43 | 12 (29.3) | 36 (83.7%) |
